# Supplementary material for: Error-Corrected Deep Targeted Sequencing of Circulating Cell-Free DNA from Colorectal Cancer Patients for Sensitive Detection of Circulating Tumor DNA
Source: Int J Mol Sci. 2024 Apr 11;25(8):4252. doi: 10.3390/ijms25084252 (PMC11049993; doi:10.3390/ijms25084252)
Supplement: Supplementary file 1 [file ijms-25-04252-s001.zip › SUPPLEMENTARY_FIGURES_S1-S5.pdf]

SUPPLEMENTARY FIGURES

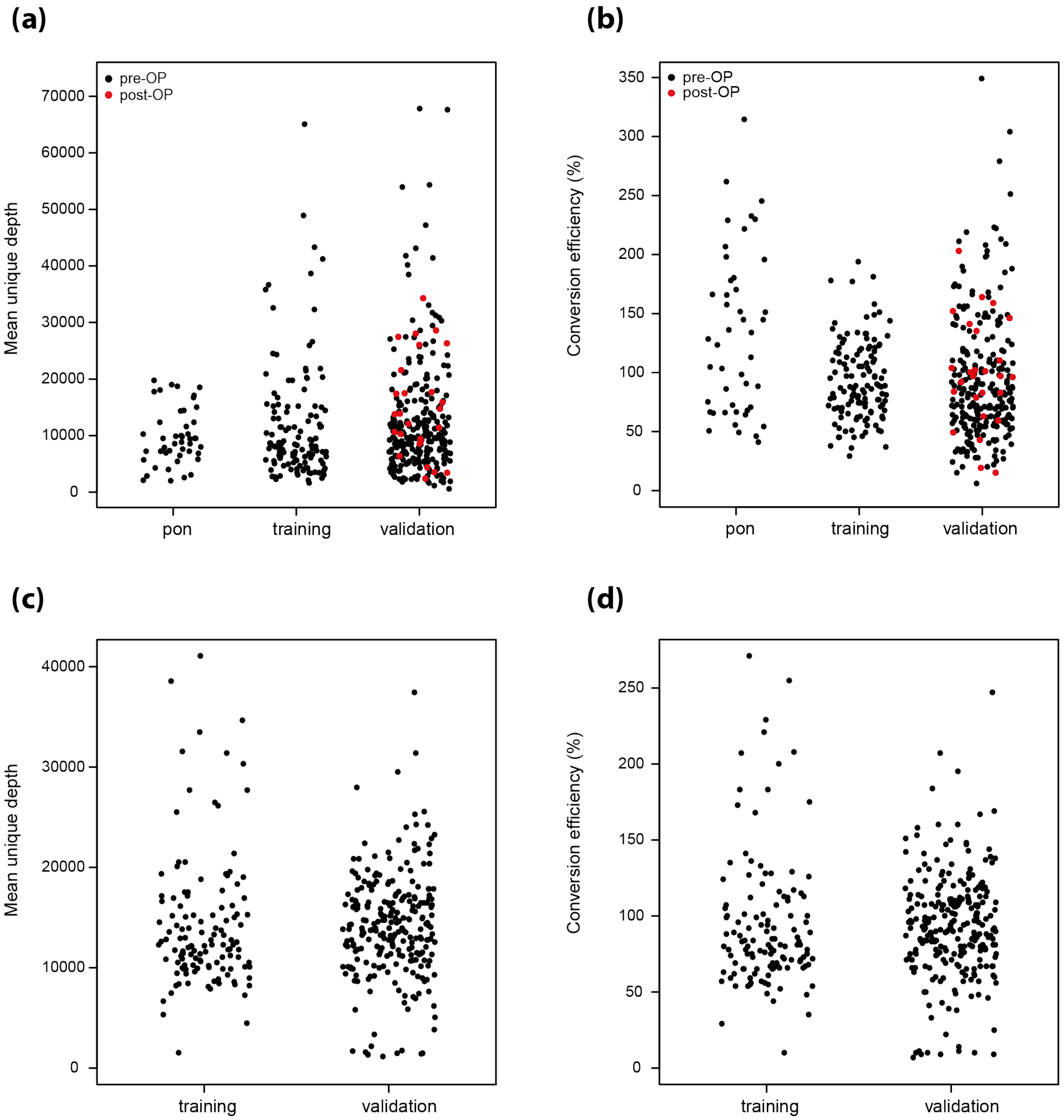

Figure S1. Coverage and conversion efficiency of plasma and PBMC samples

(**a**) Mean coverage of plasma samples in the PON, training and validation cohorts after UMI consensus generation. (**b**) Conversion efficiency (*i.e.* the mean consensus sequencing depth divided with genome equivalent used as input to library) of plasma samples in the PON, training and validation cohorts. The theoretical maximum conversion is 200%, which corresponds to where the Watson and Crick strands of all double stranded molecules of the input cfDNA have been sampled and converted into sequence data (See **Appendix A**). The post-OP samples are indicated in red. (**c**) Coverage of PBMC samples in the training and validation cohort after UMI

consensus generation. (**d**) Conversion efficiency of PBMC samples in the training and validation cohort.

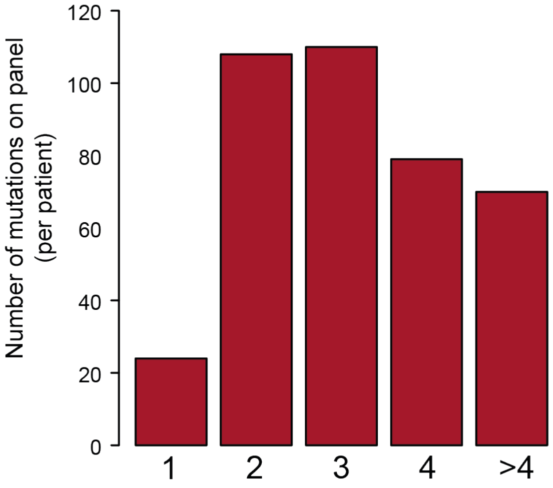

Figure S2. Mutations per patient

Total number of tumor-specific mutations (SNV, INDELs and MNVs) within the capture panel for each patient of the study (n = 381).

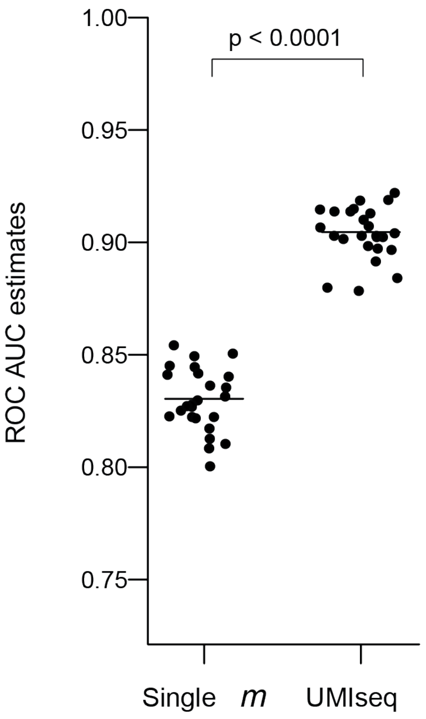

Figure S3. ROC AUC estimates for single mutation and UMIseq strategies

ROC AUC estimates for UMIseq were estimated by Monte Carlo simulations (N = 25) using the healthy controls (n = 37) and CRCs (n = 126) samples of the training cohort as described in Materials and Methods. To estimate the performance of a strategy that only uses a single mutation marker ('single *m*'), we used the mutation with the lowest *m* score (strongest mutational signal) in each plasma sample (both cancer and non-cancer control samples) directly as the sample score. The ROC AUC from each simulation was finally calculated. A student's t-test was applied to test the difference in mean ROC AUC for the single *m* and UMIseq strategy.

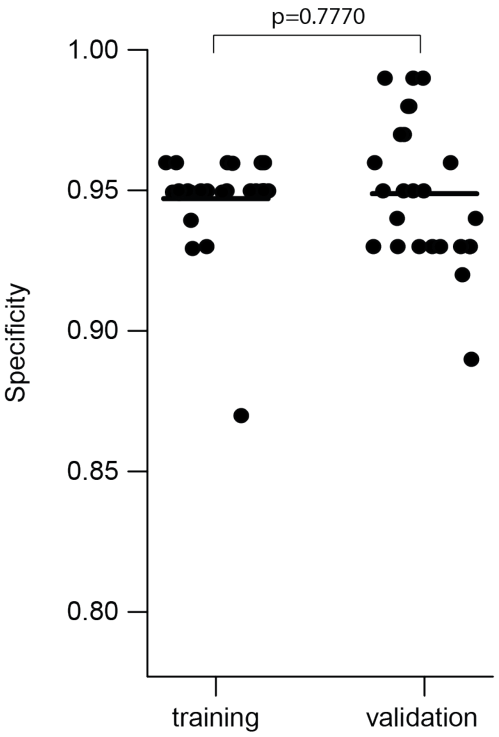

Figure S4. UMIseq specificity of the training and validation cohorts

Each point is the UMIseq specificity estimate from a Monte Carlo simulation (N = 25 in each cohort) in the healthy controls of the training (n = 37) and validation (n = 24) cohorts. For each simulation, n = 100 UMIseq scores S was calculated from random negative sample sets generated by sampling mutations from the training tumor catalog (n = 276 unique mutations) using the same procedure as described for the training of the UMIseq algorithm (see Materials and Methods). The specificity was calculated as the fraction of the 100 *S*-scores below the UMIseq threshold (*i.e.* resulted in a negative call). A student's t-test was applied to test for a difference in the mean of the training and validation specificities.

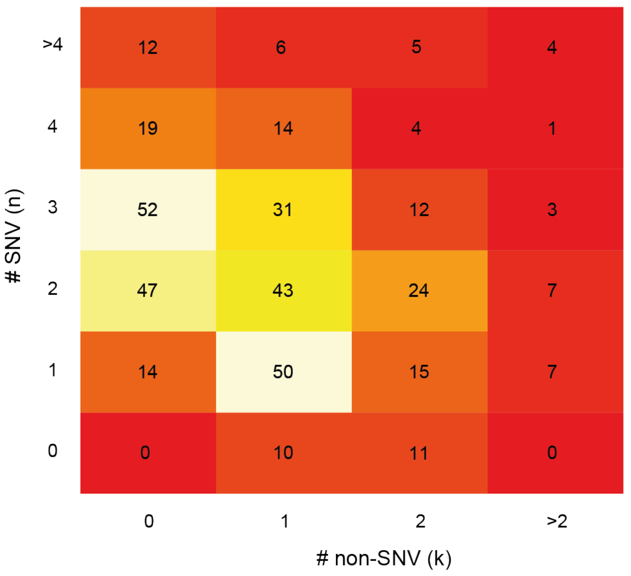

Figure S5. The distribution of SNV and non-SNV mutations

The distribution of SNVs (n) and non-SNVs (k) on the panel per patient in the study (n = 381). The individual numbers show the frequency of SNV and non-SNV combinations, as also reflected by the color scale.
